# The match between what is prescribed and reasons for prescribing in exercise referral schemes: a mixed method study

**DOI:** 10.1186/s12889-021-11094-z

**Published:** 2021-05-28

**Authors:** Colin B. Shore, Gill Hubbard, Trish Gorely, Angus M. Hunter, Stuart D. Galloway

**Affiliations:** 1 Physiology, Exercise and Nutrition Research Group, Faculty of Health Sciences and Sport, University of Stirling, Stirling, UK; 2 School of Health Sciences, Faculty of Health and Medical Sciences, University of Surrey, Guildford, UK; 3 Department of Nursing and Midwifery, University of the Highlands and Islands (UHI), Inverness, UK

**Keywords:** Prescription, Community-based research, Exercise prescription, Measurement, Physical activity, Public health practice, Surveillance

## Abstract

**Background:**

Exercise referral schemes (ERS) aim to tackle non-communicable disease (NCD) by increasing physical activity levels through prescribed exercise. However, there is a sparsity of knowledge upon what exercises are prescribed and if they are targeted towards tackling NCD.

**Method:**

Mixed methods were employed. Quantitative data was extracted from exercise prescription cards of 50 participants and were assessed for frequency, intensity, type and time of prescribed exercise. Descriptive measures of aggregate data are expressed as median (range: minimum-maximum). Thematic analysis of semi-structured interviews generated qualitative data on exercise referral instructors’ experiences of prescribing exercise.

**Results:**

Thirty-eight different types of exercise were prescribed. Median prescription was 4 (1–11) exercises per session, at a moderate intensity. Participants were prescribed a median of 35 (5–70) minutes of aerobic exercise per referral session. Exercise referral instructors prescribed exercise to improve activities of daily living, promote independence and autonomy of participants, rather than explicitly targeting the referral condition.

**Conclusions:**

Knowledge that prescribed exercises are not explicitly targeted to the referral condition provides critical information in understanding the purpose of exercise prescription. Future evaluations of ERS should be mindful of this, that is, perceived outcomes might not match up to what is being prescribed within ERS.

**Supplementary Information:**

The online version contains supplementary material available at 10.1186/s12889-021-11094-z.

## Introduction

Prolonged and sustained physical activity (PA) has been shown to positively help reduce the risk of many chronic non-communicable diseases (NCD) [1]. Exercise referral schemes (ERS) are a way to manage, prevent and treat many NCD with PA via a referral from a Healthcare professional (HCP) to an exercise referral instructor to prescribe a safe and structured exercise prescription. However, the evidence that ERS positively influence health in people who have an existing NCD is limited and short-term [2]. The prescription of ‘exercise as medicine’ is often presented as being this linear and predictable process towards health benefits [3]. However, delivery of ‘exercise as medicine’ through ERS is reliant on participants’ uptake and attendance to the ERS and adherence to the exercise prescription. Moreover, health benefits are also additionally reliant on the referral instructor’s ability to provide an appropriate prescription to address the reason for referral.

Previous research highlights that ERS are highly heterogeneous in their nature [2, 4] and descriptions of the prescribed exercise dosage are often missing, prompting a lack of clarity about the content, frequency, intensity, type, and time (FITT) of programmes [5]. One systematic review attempted to extract the type and mode of PA offered in primary studies on ERS [6]. The review was only able to go as far as reporting generic results. For example, one-to-one supervised gym-based exercise sessions, prescribed cardiovascular and resistance exercises, chair-based exercise sessions, group aerobic classes, and swimming [6]. ERS descriptions are, at best, very brief, variable and described by number of weekly sessions, duration of each session, and/or type of exercise. Therefore, key information on intensity is most often missing, meaning that a detailed description of the FITT of prescribed exercise cannot be evaluated. Moreover, without this level of detail, there is an inability to replicate the study and findings. Furthermore, a failure to establish which aspect may, or may not, have a positive or negative influence reduces the capacity to understand the dose-response or threshold that a participant must achieve to achieve any clinical benefit through the delivery of ‘exercise as medicine’. Additionally, there is paucity of evidence surrounding the process and justification that exercise referral instructors place on prescribing exercise within ERS.

Therefore, the overall aim of the present study was threefold. First, to investigate what FITT is prescribed in ERS; second, investigate any variation on exercise prescription by patient referral reason; lastly, to explore instructor views of exercise prescription.

## Methods

### Study design

The study employed a mixed-methods methodology. First, we conducted a cross-sectional analysis of routine participant data and exercise prescription cards, for individuals attending an ERS. Second, we conducted a thematic analysis of semi-structured interviews with exercise referral instructors. The University of Stirling NHS, Invasive or Clinical Research Panel approved the study (NICR (17/18) Paper No.004).

### Exercise referral scheme

At the time of the study, the ERS investigated operated in four different locations spread across Western Scotland. All four ERS locations came under the control of one leisure Trust, established by the local council and operated as a charitable company. As is common with ERS programmes, adults aged 18 years or above, who were not meeting PA guidelines as judged by a HCP, and/or were suffering from a medical condition that could potentially benefit from targeted exercise were referred to the ERS. Participants enrolled in a 12-week programme, which allowed access for two sessions per week, held on Tuesdays and Thursdays. The cost of the programme was £3.90 per session. Participants who completed all referral sessions and progressed from the programme were offered the opportunity to join the leisure centre via a concessionary membership**.** Exercise referral instructors had to hold a minimum level 3 exercise qualification and a level 3 GP referral qualification to lead exercise referral sessions. The level 3 exercise qualification is diploma in Fitness Instructing and Personal Training. The GP referral qualification is an additional qualification designed to provide instructors with the knowledge to prescribe safe and effective exercise programming for patients with medical conditions. One instructor within the scheme was undergoing the GP referral qualification at the time of the study. They were still included within the study as they prescribed and delivered the programme in conjunction with a qualified instructor.

Participants referred by their HCP were responsible for making contact with the ERS and subsequently presenting themselves at their local leisure site with their paper referral from the HCP. Participants undertook an introductory interview with the referral instructor, during which, the instructor explained the programme, timings, price, gave a tour of the facility, and reviewed the referral condition and any potential co-morbidities. The exercise referral sessions were held in a gymnasium setting, making use of cardiovascular machines, resistance machines, free weights, or using bodyweight as a means of resistance. Subsequent sessions comprised an aerobic warm up, followed by a combination of further aerobic exercise, and or resistance exercise, and a cool down period. Each participant received a personalised prescription card and performed the exercises independently, whilst being closely monitored by the instructor. Physical capacity of the various gymnasiums restricted the number of ERS participants able to attend each session to between 8 and 15 participants per session. The gymnasiums were open to the public while ERS sessions were held.

### Recruitment

An informal email approach was made to a Scottish ERS enquiring about interest in taking part in the research study. Upon showing interest in the study, one author (CS) attended a meeting with referral instructors and an ERS manager to outline study details.

#### Exercise prescription data

In addition to the standard introductory interview conducted by instructors, described above, ERS participants were made aware of the study being conducted. At the introductory interview, referral instructors explained to the participant the study information and allowed time for any questions. Participants were given a minimum of 48-h to provide informed consent to release their anonymised details and exercise prescription data. It was made explicitly clear that participants choosing not to be part of the study would receive the same level of care, i.e. no change to their experience of ERS. Those who chose to be a part of the study signed informed consent forms that were counter signed by an exercise instructor. The nature of the study required no new data to be collected and made use of data routinely held by the scheme. Participants who were referred to, and took up referral in, the scheme between June 2018 and December 2018 were invited to take part in the study.

#### Exercise referral instructor interviews

Participant information sheets were distributed to ERS instructors at the initial study information meeting, allowing instructors to ask questions surrounding the project. Instructors were provided with email and phone contact details, if they had further questions. Instructors were then invited to participate in the study; if they agreed, a suitable time and date was set for one author (CS) to visit the referral sites to conduct the interview. Before commencement of interviews, ERS instructors were provided with a brief verbal recap of the purpose and format of the interview, alongside assurances of confidentiality and a further opportunity to withdraw if required. All ERS instructors provided verbal and written informed consent for digital audio (Olympus VN-731PC) recording and use of anonymised quotations. One hundred percent (*N* = 6) of referral instructors employed at the time of the study agreed to participate.

### Data extraction and objective measures

#### Exercise prescription data

Data was extracted from two documents held by the ERS: 1) referral forms that were pre-populated by the HCP to the ERS; and 2) prescription cards that included free text information detailing the individualised prescribed exercises for participants to follow. An exercise referral instructor at the relevant referral site collected pre-populated referral forms. Referral forms were photocopied and participants’ name, address, date of birth, referring HCP’s details and any data unrelated to the study, were redacted. Prior to removing the address details, postcodes were converted into indices of deprivation quintiles via the Scottish Government’s official index of multiple deprivation tool for measuring indices of deprivation (SIMD) [ 7]. The same anonymising process was completed for exercise prescription cards. Free text data on the prescription cards amounted to the following: date(s) of session(s), prescribed type of exercise (e.g. rowing machine), prescribed time duration (minutes) of aerobic exercises, prescribed speed at which to complete aerobic exercise (e.g. 2.1kph), prescribed mass (kg) to lift of resistances exercises, and prescribed number of repetitions and sets of each resistance exercise. An example of a prescription card is available within the supplementary material (supplementary material 1).

Descriptive variables extracted were gender, age, indices of multiple deprivation, reason for referral to ERS and co-morbidities. Age was recorded in years on the day of obtaining exercise referral membership, and grouped into the following year bands: 16–24, 25–34, 35–44, 45–54, 55–64, 65–74 and 75+ [8]. Indices of multiple deprivation were recorded between one (living in most deprived areas) to five (living in least deprived areas). Reason for referral were grouped into seven categories: neurological, frailty and mobility, musculoskeletal, cardiovascular, general fitness, mental health, and obesity. While general fitness was not a medical condition, it was a term listed by HCP’s as a reason for referral. Co-morbidities were defined as the number of additional medical conditions that participants made referral instructors aware of during the introductory interview, before commencing the prescribed programme.

Definitions of FITT that were used to report prescribed exercise, extracted from prescription cards are shown in Table 1. Traditionally, frequency refers to how often a person will perform exercise (per day, per week) [9]. However, for the purpose of this paper, attendance, or how often a person exercises, were represented by the term ‘session count’ [10]. Date(s) of sessions provided a measure of when the referral session occurred and were used as an objective measure of attendance. Therefore, frequency was adapted to represent the number of exercises completed within each exercise referral session. Intensity: Where applicable, the prescribed speed of aerobic exercise was matched against compendium of physical activities, providing metabolic equivalent of task (METS) as a reference value. Light-intensity aerobic activity was defined as an activity done at 1.1 to 2.9 METs, moderate-intensity activity was defined as 3 to 5.9 METs, while vigorous activity was an activity defined as ≥6 METs [11]. Subsequently, light intensity, moderate and vigorous activity were assigned values of one, two and three, respectively, and used as a measure of exercise intensity. Further, intensity value (1–3), was multiplied by the duration of activity (in minutes) to create a measure of total aerobic exercise load [12]. A similar process was used to create a resistance-training exercise load, which was the multiplication of mass lifted (kg), sets completed, and repetitions per set. For example, chest press 10 kg × 10 repetitions × 2 sets = resistance training load of 200 kg. Furthermore, total lifted load mass (kg) was the sum of all resistance-training loads completed per session. Time: The duration of time the participant performs an exercise and expressed in minutes. Type: The category of exercise performed and expressed as aerobic or resistance exercise. Additionally, magnitude of progression between sessions was expressed as the difference between loads completed in the first recorded exercise session, and the last recorded exercise session. Magnitude of progression between sessions was reported as increase or decrease of the following measures: frequency count of total number of exercises completed per session; frequency count of either resistance or aerobic exercises completed per session; time duration (minutes) of aerobic exercises completed; and total lifted load mass (kg) per session. Magnitude of progression between sessions was used to evaluate adaptations to the exercise stimulus noted by the exercise instructor.

**Table 1:** Definitions of FITT used to report the prescribed exercises extracted from ERS prescription cards

Component	
Frequency	The number of exercises completed within each exercise session.
Intensity	Predetermined value matched against compendium of physical activities and respective MET’s indicating effort to perform activity.
Time	The length of time the participant performs an exercise. Expressed in minutes.
Type	The category of exercise performed. Expressed as aerobic or resistance exercise.
Aerobic	Predominant focus of the exercise performed is to improve cardiovascular conditioning. Examples of such exercises can include treadmill walking, jogging, recumbent cycle or up-right cycle bike.
Resistance	Predominant focus of the exercise performed is to improve muscular strength via the use of free weights, resistance machines or body weight. Examples of such exercises can include chest press, bicep curl, and sit-to-stand.
Repetition	One complete motion of a resistance exercise, measured as a whole number.
Sets	A group of consecutive repetitions measured as a whole number.

#### Exercise referral instructor interviews

In addition to exploring exercise instructors’ perceptions of prescribing exercise, the interview also explored exercise instructors’ perceptions of motivating participants to uptake and attend the programme and adhere to the prescription. However, this manuscript will only address exercise instructors’ perceptions of prescribing exercise. Given the exploratory and inductive focus of the research on exercise instructors perceptions of prescribing exercise in ERS, face-to-face, semi-structured interviews were undertaken. Interviews were conducted at the ERS sites where instructors worked, in a quiet meeting room. The interviews were guided by a semi-structured schedule (supplementary material 2). The schedule was developed following a literature review and collaborative discussions among the authors. Initial pilot testing of the questions was conducted with an exercise instructor who was not a study participant, but had experience of prescribing exercise to clinical populations. The guide was not designed to have questions posed chronologically but rather, in an order that seemed to follow the natural flow of the conversation. The schedule used open-ended questions, probing topics of interest to the study, whilst allowing discussion of issues of importance to the interviewee. Use of open-ended questions allowed instructors the opportunity to express their experiences, providing deeper and detailed insights into their experiences. Median length of Interviews was 44 min (range: 37–53 min).

### Analysis

#### Exercise prescription data

Analyses were performed using Statistical Package for the Social Sciences (SPSS) version 23 (SPSS Inc., Chicago, IL, USA). Exploratory analyses were undertaken to establish descriptive measures of all variables; age, gender, SIMD, referral reason, co-morbidities, FITT of prescribed exercise, magnitude of exercise prescription change. Aggregate data is expressed as median (range: minimum-maximum) across all participants at the four referral sites. The use of median value as a measure of central tendency is deemed appropriate for skewed data. Further descriptive measures are reported across four individual sites (site A, B, C, D) for distribution of participants, referral condition, count of sessions and time and lift load. Last, Moods Median allows for a statistical analysis of any relationship between referral condition and the prescribed exercise across sites A, B, C and D. Moods Median allows analyses of two or more categories within nominal independent variables. In this instance, it allowed the seven-referral conditions to be examined. Statistical significance was set at *p* ≤ 0.05.

#### Exercise referral instructor interviews

Data were transcribed verbatim. Transcripts and sound files were stored in an encrypted research drive, held at the University of Stirling. An inductive thematic approach to analysis was adopted [13]. Analysis were performed using three-steps. First, an initial set of codes were set by identifying recurring words within the dataset, or generated from words of interest to the authors. Once an initial list of codes was generated, they were cross-referenced against each other and where appropriate, combined. For example, ‘purpose’, ‘achievable’ and ‘advice’ were grouped together to create the code ‘providing knowledge and benefits to becoming active’. Generated codes related to the prescription of exercise. Transcripts were coded by hand, constantly revisited and cross-referenced, throughout this first iterative step by three authors (CS, GH and TG).

Second, coded data were grouped into six descriptive themes. First, instructor intentions within ERS, which is defined as the role that instructors perceive they have. Second, communication approaches from ERS instructor, which is defined as approaches that instructors take to motivate the participants to take-up, attend ERS and adhere to their exercise prescription. Third, behaviour change approaches, which is defined as instructors’ use of behaviour change techniques (BCTs). Fourth, instructors’ perceptions of participants’ views of ERS, which is generated from a combination of views and emotions that a participant might directly or indirectly say or demonstrate to an ERS instructor. Fifth, barriers towards providing ERS, which is defined as any situation that might hinder delivery of ERS. Last, success of ERS, which is defined as what instructors valued as an outcome for participants, or instructors’ perceptions of what participants valued.

Third, all codes under the six descriptive themes were analysed in the context of the authors understandings and interpretation of the topic of prescribed exercises. Two authors (CS and GH) developed the following conceptual framework, basing it upon the six descriptive themes and codes. The conceptual framework comprised one interpretative theme: purpose of exercise prescription. Throughout all three-stages described above, draft analyses were circulated between three authors (CS, GH, TG). Face-to-face meetings allowed discussions about initial coding, descriptive themes and thereby reaching consensus on descriptive thematic analysis and interpretation.

## Results

### Exercise prescription data

#### Participants

Fifty participants agreed to participate in the study. Just over half of the participants were female (52%), median age of 70 years (26–83) and predominately over 55 years of age (76%). The majority of participants resided in areas classified as deprived (36% SIMD 1–2 combined, 36% SIMD 3, 28% SIMD 4–5 combined). General fitness and cardiovascular disease were the two most common reasons for referral, but all referral reasons were represented (Table 2). Participants presented at the ERS with a median of 2 (0–5) comorbidities.

**Table 2:** Frequency count and percentages of participant variables at each of the four ERS sites. Total sample size was *n* = 50

Participants		Frequency (N)	Percentage (%)
Gender	Female	26	52
	Male	24	48
Grouped age (y)	25_34	3	6
	35_44	2	4
	45_54	7	14
	55_64	9	18
	64_74	20	40
	75+	9	18
Referral reason	Neurological	2	4
	Frailty and mobility	2	4
	Musculoskeletal	6	12
	Cardiovascular	15	30
	General Fitness	18	36
	Mental Health	4	8
	Obesity	3	6
Scottish index of multiple deprivation	Most Deprived	7	14
	More Deprived	11	22
	Deprived	18	36
	Less Deprived	12	24
	Least Deprived	2	4

#### Session count, frequency, intensity, time and type of prescribed exercise

Across all sites, participants median session count was 8 (1–25), with males (10, 1–25) undertaking slightly more referral exercise sessions than females (8, 1–21). Across the programme, thirty-eight different types of exercises were prescribed, of which eight were aerobically focused and thirty resistance-based (Table 3).

**Table 3:** Classification and type of exercise prescribed across referral scheme

	Targeted area	Exercises as described on prescription card
Aerobic	Cardiovascular system	Walking, Cross trainer, Rower, Treadmill, Recumbent bike, Hydro, Up-Right bike, Unspecified / Exercising alone
	Upper-body musculature	Arm raises, Fly, Chest press, Bicep curl, Lateral pull down, Upper back, Shoulder press, Barbell curl, Dumbbell front raise, Cable pull down, Triceps dumbbell kickbacks, Bent over row, Seated row, Lateral raises, Wall press
Resistance	Trunk musculature	Torso rotation, Hip Hinge, Crunch, Donkey kicks
	Legs musculature	Sit to stand, Heel taps, Weighted step ups, Hamstring curl, Lunge and lateral raise, Calf raises, Deadlift, Leg extension, Leg curl, Leg press
	Other	Unspecified circuit

#### Aerobic frequency, intensity, time and type

Median frequency of exercises per referral session was 4 (1–11), of which, participants were prescribed 2 (0–5) aerobic based exercises per session. The ability to determine intensity for aerobic exercises was classified for six exercises, all of which were prescribed to be performed at a moderate intensity; only jogging / running on the treadmill was prescribed and performed at light or vigorous intensity levels. The calculated median aerobic load across the programme was 70 (10–140). Across all sites, participants were prescribed a median of 35 (5–70) minutes of aerobic exercise per referral session, with the rest of the time taken up by rest periods or resistance exercises. Jogging on the treadmill and up right bike were the two most prescribed aerobic exercises (Table 4). Data upon intensity, time and aerobic load was unavailable for two exercises, unspecified / exercising alone and hydro.

**Table 4:** Total frequency count and median (min-max) of intensity, time, and training load values, of aerobic exercises prescribed across four ERS sites

Type	Frequency	Intensity ^a^	Time duration (mins)	Aerobic training load ^b^
Unspecified / exercising alone	2	*	*	*
Hydro	5	*	*	*
Treadmill	380	2 (1–3)	15 (1–35)	30 (2–70)
Up right bike	339	2 (2–2)	15 (5–45)	30 (10–90)
Recumbent bike	246	2 (2–2)	15 (5–30)	30 (10–60)
Rower	207	2 (2–2)	10 (3–20)	20 (6–40)
Cross trainer	68	2 (2–2)	7 (5–11)	14 (10–22)
Walking	8	2 (2–2)	5 (5–10)	10 (10–20)
Median of total aerobic data	2 (0–5)	2(1–3)	35 (5–70)	70 (10–140)

*Data unavailable

^a^ Intensity is classified as follows; 1 = low, 2 = moderate, 3 high

^b^ Aerobic training load is calculation of intensity x duration

Figures in parenthesis = range

#### Resistance frequency, intensity, time and type

Participants were prescribed a median of 1 (0–9) resistance-based exercises per session. Explicit intensity of resistance exercises prescribed was not evident on prescription cards, however, the total lift load mass is reported as proxy. Participants were prescribed a median of 10 (1–20) repetitions and 2 (1–7) sets, per resistance exercise, per session. Chest press and lateral pull down were the two most prescribed resistance-based exercises, accounting for 15 and 12% respectively, of total prescribed resistance exercises (Table 5). Exercises focusing on the musculature of the upper body accounted for 59% of total resistance prescription; exercises focusing on leg musculature comprised 35% of total resistance exercises and trunk musculature and other (circuits) were 3% each.

**Table 5:** Total frequency count and median (min-max) values of sets, repetitions, mass per rep and lifted load per session, of resistance focused exercises prescribed across the four ERS sites

Type	Frequency	Median Sets	Median Repetitions	Median Mass per rep (kg)	Median Total Lifted load
Unspecified circuit	44	*	*	*	*
Arm raises	8	4 (3–4)	9.5 (8–12)	*	*
Fly	2	1.5 (1–2)	10 (10–10)	10 (10–10)	150 (100–200)
Chest press	202	2 (1–4)	10 (1–15)	12.5 (5–30)	300 (10–1440)
Bicep curl	90	2 (1–7)	10 (8–12)	4 (2–8)	98 (20–384)
Lateral pull down	165	2 (1–4)	10 (7–15)	20 (10–35)	500 (100–1540)
Upper back	4	2.5 (2–3)	11 (10–12)	16.25 (15–18)	442.5 (360–525)
Shoulder press	65	3 (2–4)	10 (8–12)	6 (2–15)	192 (40–500)
Barbell curl	17	*	*	*	*
Dumbbell front raise	36	2 (2–3)	12 (10–12)	1.5 (2–5)	36 (30–120)
Cable pull down	12	2.5 (2–4)	10 (10–15)	11.25 (7–16)	303.75 (135–780)
Triceps dumbbell kickback	23	2 (2–1)	10 (10–10)	7.5 (8–10)	150 (100–150)
Bent over row	23	2 (1–3)	10 (10–12)	10 (1–20)	300 (30–720)
Seated row	42	2 (1–2)	10 (1–15)	15 (10–20)	300 (20–480)
Torso rotation	11	1 (1–1)	10 (10–10)	1 (1–3)	10 (10–25)
Lateral raises	86	2 (1–3)	10 (6–12)	2 (1–12)	48 (10–144)
Leg press	72	2 (1–3)	10 (10–15)	25 (1–40)	450 (10–1200)
Leg curl	44	2 (1–2)	15 (10–20)	15 (5–25)	400 (77–600)
Leg extension	72	2 (1–4)	12 (10–20)	10 (5–35)	360 (75–750)
Dead lift	24	2 (1–4)	12 (10–12)	15 (10–20)	360 (150–720)
Calf raise	45	2 (1–2)	16 (10–20)	*	*
Lunge lateral raise	16	2 (2–3)	11 (1–12)	2 (1–12)	60 (24–360)
Hamstring curl ball	30	2 (1–3)	12 (1–15)	*	*
Step up	72	2 (1–4)	12 (1–24)	4 (4–5)	96 (5–120)
Heel taps	9	1 (1–1)	10 (10–10)	*	*
Crunch	15	1 (−2)	15 (10–15)	*	*
Sit-to-stand	81	2 (1–4)	10 (1–15)	5 (1–10)	120 (10–336)
Wall press	10	1.5 (1–2)	12 (10–15)	*	*
Hip hinge	6	3 (1–3)	10 (1–12)	12 (8–16)	360 (8–432)
Donkey kicks	7	1 (1–3)	16 (12–20)	*	*
Median of total resistance data	1 (0–9)	2 (1–7)	10 (1–20)		1224 (60–4728)

*Data unavailable

Figures in parenthesis = range

**Fig. 1:**
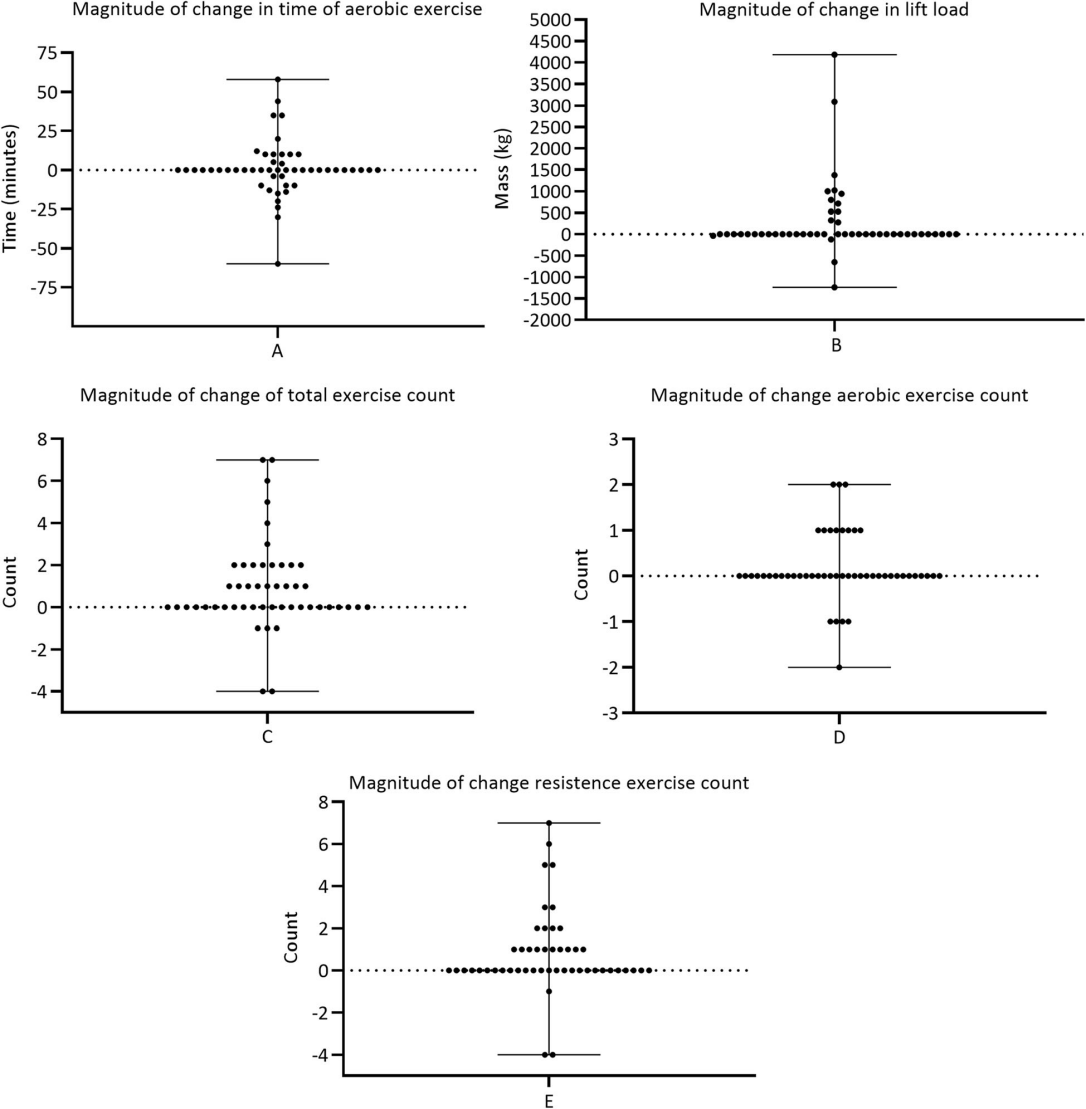
Assessment of magnitude of change expressed as difference between measures at first recorded exercise session and same measure at last recorded session. Data expressed as median (min to max)

As participants progressed through the programme, magnitude of prescription change is reported accordingly, between first and last session completed. This data is aggregate and inclusive of participants who completed one session, the median of eight sessions and the small minority of those who completed above 20 sessions. Median change across aerobic time (see Fig. 1A) and total lifted load (kg) of resistance exercise (see Fig. 1B) were 0 (− 60–58) and 0 (− 1240–4181) kg, respectively. Zero median changes were reported in total exercise count (see Fig. 1C) across participants (0, − 4-7). Further, zero change in median count value of aerobic exercises performed 0 (− 2–2) were observed (Fig. 1D) and count of resistance exercises performed 0 (− 4–7) (Fig. 1 E).

Site D (*N* = 32) saw the largest number of participants attending, compared with sites C (*N* = 9), A (*N* = 5) and B (*N* = 4) (Table 6). Site D had the largest number of participants whose primary referral reason was for a cardiovascular condition (86%). Site D saw participants being prescribed the greatest number of aerobic based exercises (1–7); prescribed more time on aerobic activities (43, 18–54), and prescribed the fewest number of resistance exercises per session (1–4) and lowest total lifted load (736 kg, 190–1442) per session. Participants at sites B and C performed greater total number of exercises per session. The two sites with highest prescribed lifted loads (A and C) prescribed almost less than half the time in aerobic activities than site D.

**Table 6:** Percent of participants’ based on referral condition and median (min-max) total exercise count, count or aerobic and resistance exercise, time spent in aerobic exercise and lift load of resistance exercises, prescribed at four different referral sites

	Site A	Site B	Site C	Site D
Neurological	50%		50%	
Frailty and mobility		50%	50%	
Musculoskeletal			50%	50%
Cardiovascular		7%	7%	86%
General Fitness	17%	11%	11%	61%
Mental Health	25%			75%
Obesity			33%	67%
Number of exercises per session	5 (4–10)	7 (5–8)	7 (4–8)	3 (1–7)
Number of aerobic exercise per session	1 (1–2)	2 (1–2)	2 (1–2)	3 (1–7)
Number of resistance exercises per session	4 (2–8)	5 (3–7)	5 (2–7)	1 (1–4)
Time spent performing aerobic activities (min)	25 (5–30)	39.5 (23–45)	20 (14–24)	43 (18–54)
Total lift load per session (kg)	1799 (765–2833)	936 (830–1078)	1727 (515–3318)	736 (190–1442)

Figures in parenthesis = range

Moods Median revealed no significant difference between referral condition and count of prescribed exercise per session (χ2 (6) = 3.70, *p* = .71), number of prescribed resistance exercises per session (χ2 (6) = 7.28, *p* = .29) and prescribed total lifted load per session (χ2 (5) = 7.54, *p* = .18). Statistical significance was observed for referral condition and time spent performing aerobic activity (χ2 (6) = 14.80, *p* = .02) and between referral reason and number of aerobic exercises performed (χ2 (6) = 20.01, *p* = .003).

#### Exercise referral instructor interviews

**Fig. 2:**
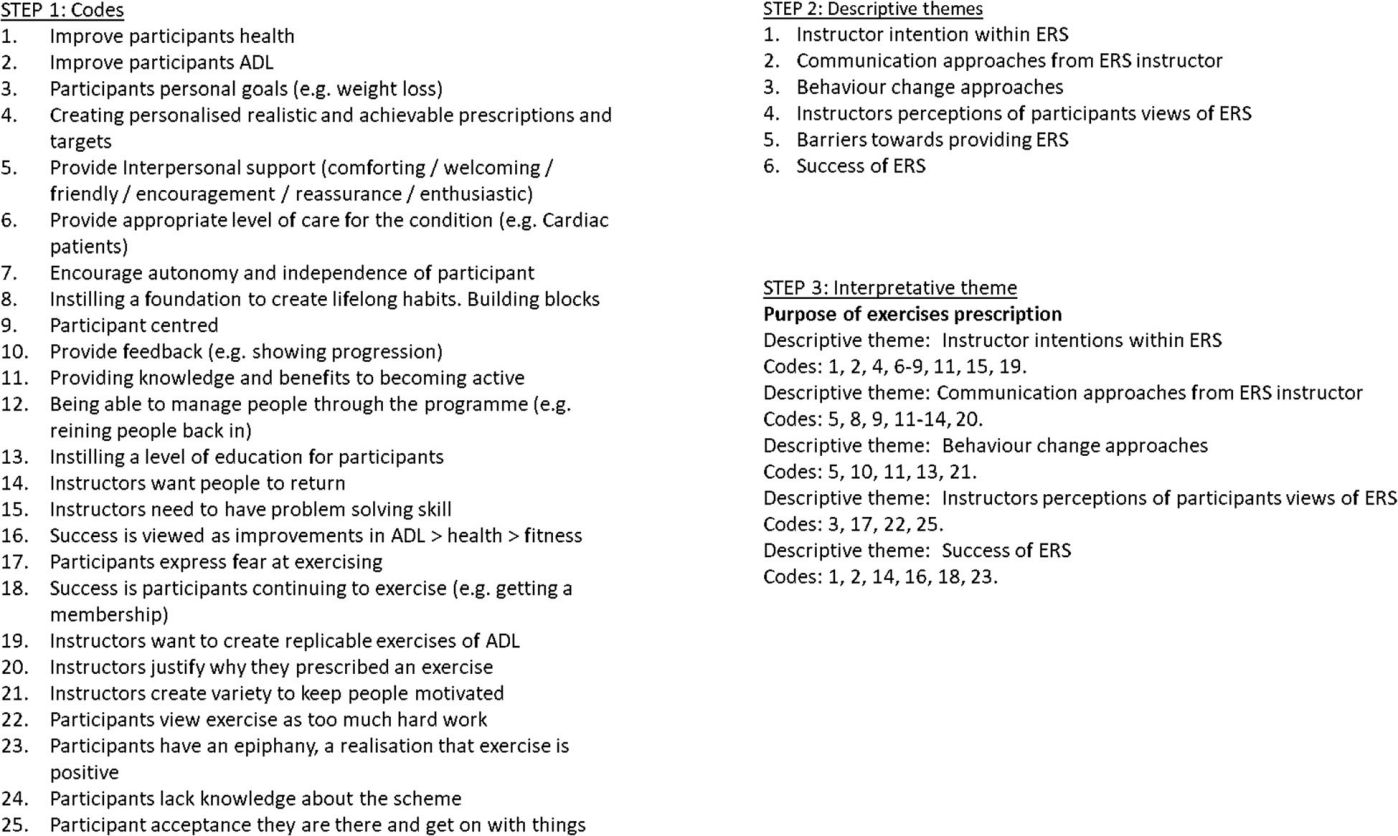
Three step coding framework of semi-structured interviews of exercise referral instructor’s experiences or delivering exercise prescription

In total six interviews were conducted. Fifty percent of instructors were female, with a median of 3 (1–12) years of experience working as an exercise referral instructor. Twenty-five initial codes were created. These codes were grouped into six descriptive themes. Descriptive themes are understood under one conceptual theme, the justification and purpose of exercise prescription (Fig. 2).

#### Purpose of exercises prescription

Instructors provided a mixed response when asked if the objective of ERS was to improve health or fitness. Acknowledging that participants were referred for a health condition, instructors described that fitness and health go hand-in-hand with each other.

‘Yeah, I would say a bit of both. […] being a health and fitness professional is you want to get people more active, regardless of what condition they may have at the time, but yeah, just I would say just in general, get a bit more active’ (In5).

‘I think with the people that we work with, the majority of them, health is the motivation for referring them, and you can see that through from whatever reason […] some of them will say that, oh, I feel much fitter for it, but the majority of them just want to feel better’ (In1).

Unanimously, instructors said that supporting participants to be independent and be able to carry out activities of daily living such as, tying their own shoelaces was the main goal of ERS and therefore the prescription. Therefore, instructors prescribed exercises that either mirrored activities of daily living or would have a positive influence on activities of daily living; ultimately providing holistic benefits for the participant. Instructors described how participants expressed success of ERS as improvements in their (participants) activities of daily living. Instructors continued to describe that prescribing exercises that were simple, replicable at home, exercise that help promote and give autonomy and independence back to participants.

‘I’ve managed to do this, tie my shoelace. Things like that. I mean, you can see the look on their faces to actually get a bit of independence back in their lives which is my main goal for it. To give them back that independence and maintain it’ (In4).

‘I can climb the stairs with shopping bags, it’s just so much easier, I can’t believe the difference it’s made’ (In1).

‘we want them to be independent, because it’s a lifestyle change, it’s not just […] well done, see you later’ (In5).

Within the initial interview with participants, instructors established any potential barriers or facilitators to undertaking the programme and discussed with the participant any exercises they would like to undertake, or any specific goals from participation in the scheme. Instructors described they need a degree of flexibility when prescribing an exercise programme. For instance, having to deal with participants who are negative, working around clinical limitations of referral condition and co-morbidities, participant’s likes and dislikes and availability of exercise equipment.

‘what do you fancy starting on today, so it’s not always just us telling them what they’re going to do, so we try and be quite laidback, quite flexible. We’re quite positive, because some of them are very negative, so we will always try and put a positive slant on things’ (In1).

‘depends on again the individual, what they’ve been referred for, what their previous activity is like as well, take all those sort of things into consideration. But I always tend to start off low rather than starting off too high and maybe having to regress it, it can demotivate them’ (In6).

## Discussion

Due to specificity of adaptations to an exercise stimulus, the FITT of an exercise prescription is usually dependent on the type of outcome desired [14]. For instance, improving balance and reducing risk of falls vs. lowering blood pressure would each require a different activity focus [15, 16]. Therefore, targeting exercise prescription to a specific referral condition will undoubtedly have a greater impact. The FITT of an exercise prescription should contain a combination of aerobic and resistance-based activities. Exercise intensity should progress from low or moderate to higher intensities as adaptations take place. Sessions should occur, as a minimum, on 2–3 days per week, preferably more, and last between 30 and 60 min per session. Dependant on outcomes desires, interventions should last for at least six months [14]. Therefore, in theory, the prescribed FITT within ERS should be twofold: first, personalised to the medical referral reason, and second, contain structured elements that can be progressed. However, exercise prescription within the reported ERS does not appear to conclusively match such an approach.

The present study reports that participants sessions are between 30 and 60 min per session; and participants had the opportunity to attend two times per week as part of their ERS membership. The prescribed exercise did cover a broad range, however, four exercises (treadmill, up right bike, chest press, and lateral pull down) accounted for 42% of the total exercise prescription. Intensity does start off at a low-moderate intensity, however, there is limited evidence to suggest that over the prescription that there was a magnitude of change or progression. Moreover, as the session count was low and in keeping with many previous studies that have reported attendance at ERS [17, 18, 10]; it may be unlikely to observe a change in magnitude of the prescription.

This study found no evidence to suggest exercise were targeted to certain medical referral reasons. Taken on this data alone, it is suggestive that ERS prescription is not fit for purpose. Currently ERS effectiveness is judged upon a ‘*disease centred*’ approach. That is, the focus on a clinical outcome or improvements of PA. However, as evidenced from the instructor interviews, the studied ERS does not operate in such a manner. Therefore, if effectiveness of ERS continue to be assessed on broad clinical referral or improvements in PA, ERS and its subsequent prescription will be seen as ineffective and not fit for purpose. Therefore, there is a need to understand the multidimensional nature and concept of success, which may prompt focus upon additional measures of impact of ERS, such as measures of quality of life or activities of daily living [19, 20]. Previous work has demonstrated that the impact of ERS is felt more holistically [20]. Mills and colleagues recommended the need to develop alternative indicators (e.g., social benefits) for a more representative evaluation of ERS [20]. This would bring ERS in line with the health service in following a patient centred approach and be a truer reflection of what is prescribed and why [21].

This study reports that instructor’s value and are aware of trying to improve patients’ clinical condition. However, with a lack of evidence that demonstrates exercises are prescribed to a certain condition; creating independence, autonomy and supporting daily activities is the purpose of the prescription. Instructors actively prescribe exercises that would resonate with participants, due to the perceived perceptions that participants value the associated outcomes of improvements in their daily activities (e.g. tying shoelaces, carrying shopping or walking up the stairs without being out of breath). Instructors described their aim was to encourage participants to adopt a long-term physically active lifestyle. In providing a prescription, co-created with the participant, that is achievable and fun. Instructors perceived this as having a greater long-term impact. This sits in line with previous research that reported that if participants could relate to the exercise or PA, they were more likely to engage with their exercise prescription [22].

ERS has previously been seen as a non-essential service by public health commissioners, being deemed too costly a ‘medicine’ to fund from the public purse [3]. However, at a policy level, schemes like ERS are promoted as a potential panacea to the problem of rising inactivity levels and associated increased prevalence of NCD. This disconnect between ERS as a panacea for health and being viewed as a non-essential service may be reflective of different perspectives held of ERS. Those delivering the programme are acutely aware of their role to help the individual through their prescription. Meanwhile, it has been reported that commissioners often see ERS as a scheme that will have influence at a population level [3]. The lack of a shared understanding of the purpose of ERS has been discussed elsewhere [20] albeit, using different methodology. We share the views of Mills et al. in the need for a sharing of aims and objectives between the stakeholders and indeed for the fitness industry to apply evidence based practice in their exercise prescription programming. However, it appears this has still not been addressed in policy or practice. Therefore, it unsurprising that there is no national policy [23] or best practice on what should be prescribed within ERS.

### Strengths and limitations

The present study benefitted from a naturalistic approach by extracting handwritten data from prescription cards and interviewing the instructors who prescribed and delivered the session. Prescription card data supports the validity of the study, in that it provides a realistic account of FITT of prescribed exercise. The FITT of exercise prescription is commonly unreported, hence, a strength of this study is a description of FITT within an ERS. Further strengths include a high response rate with 100% of referral instructors employed at the sites participating the study. Therefore, views expressed reflect those of all the exercise referral instructors, not just a proportion. There are, however, several limitations. Data were collected from a small ERS and despite obtaining 100% of referral instructors employed in this ERS, the cohort is small representative operating with limited opportunities. Subsequently, the present study is limited in conferring such findings on to larger ERS, which may have more facilities and opportunities. The present study lacked the methodology and logistics to explore adherence to the prescribed exercise. Due to the low number of participants per sessions, instructor confirmed that participants fully adhered to the prescription. However, this is anecdotal, and we are not able to confer that adherence was 100% of the prescription. Consideration of reliability relates to one author (CS) who conducted the interview data collection is a limitation. Due to logistical restraints around funding of an independent researcher to conduct the interviews, it was not possible to mitigate for any researcher bias around conducting and interpretation of interview data.

## Conclusion

Knowledge of the dose of exercise prescribed and its justification within ERS provides critical information in understanding what exercise is prescribed and whether ERS is tailoring exercise prescriptions towards tackling NCD. However, the evidence base demonstrates that prescribed exercise is limited in its scope and does not differ between referral conditions. Rather, exercise referral instructors prescribed exercise to improve activities of daily living, to promote independence and autonomy with the participants. Subsequently, this could in part contribute to ERS schemes being deemed ineffective. This suggests that research, policy makers and stakeholders should readdress its measure of ERS effectiveness and to consider the purpose of the prescribed exercise.

## Supplementary Information

**Additional file 1: Supplementary data 1**. Example exercise prescription card. **Supplementary data 2**. Semi-structured interview schedule and prompts for exercise referral instructors.

## Data Availability

The data that support the findings of this study are available from the corresponding author on request.

## Abbreviations

ERS: Exercise referral scheme
HCP: Healthcare professional
NCD: Non-communicable diseases
PA: Physical activity
