# Supplementary material for: The match between what is prescribed and reasons for prescribing in exercise referral schemes: a mixed method study
Source: BMC Public Health. 2021 May 28;21:1003. doi: 10.1186/s12889-021-11094-z (PMC8161921; doi:10.1186/s12889-021-11094-z)
Supplement: Supplementary file 1 — Additional file 1: Supplementary data 1. Example exercise prescription card. Supplementary data 2. Semi-structured interview schedule and prompts for exercise referral instructors. [file 12889_2021_11094_MOESM1_ESM.docx]

**Supplementary data 1**. Example exercise prescription card


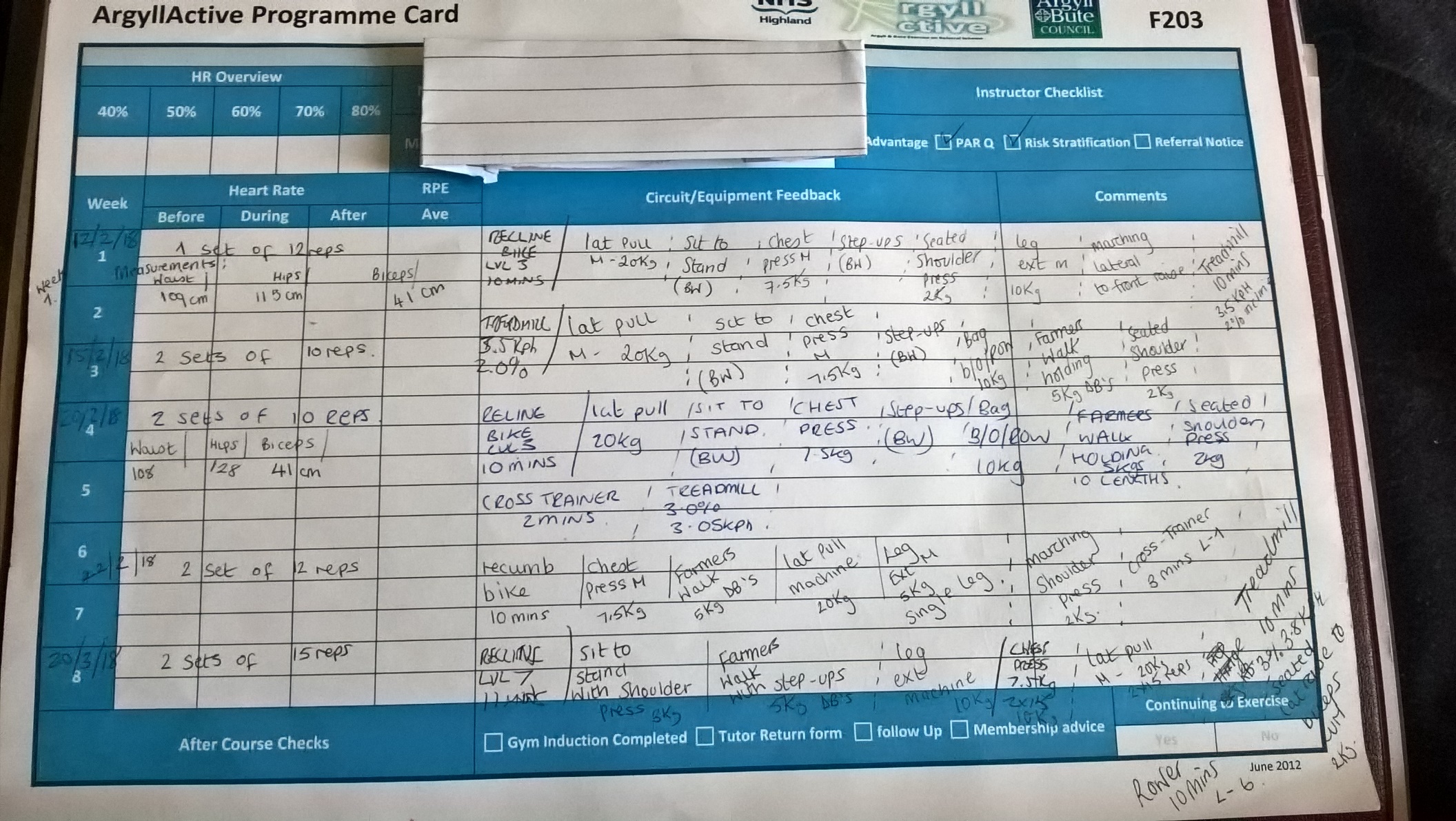


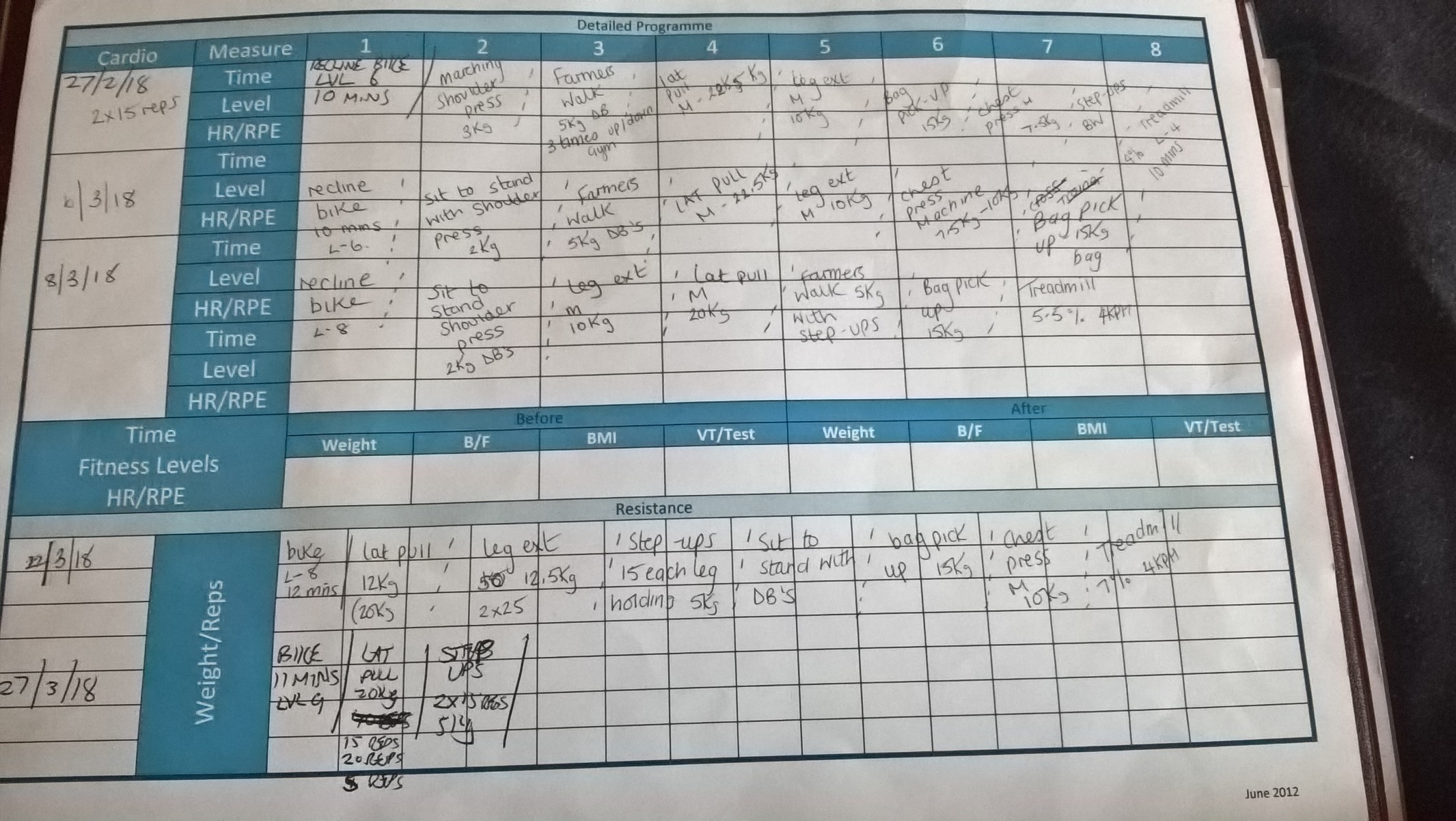


**Supplementary data 2**. Semi-structured interview schedule and prompts for exercise referral instructors

| **Topic area** | **Questions** | **Prompts if and when appropriate** |
| --- | --- | --- |
| **Demographics of professional** | 1. Age 2. How many years of experience do you have as an exercise referral instructor? 3. Can you tell me what qualifications you hold as an ERS instructor? 4. Do you have any additional qualifications that you deem relevant to your job as an exercise referral instructor? | Can you give me examples of this?  Can you share a time that happened?  Q2 – years as non-exercise referral instructor  Q4 – How does that qualification help you? |
| **Role of an ERS instructor in prescribing exercise** | 1. Can you explain to me what you see your role as, in regard to exercise referral? 2. Putting yourself in the shoes of the client for a second, can you talk me through what clients might see exercise referral as / see you as 3. Can you tell me how does your role differ between clients? 4. Can you tell me how diverse your role with the client is - Does it expanded away from just the referral? | Can you give me examples of this?  Can you share a time that happened?  Q1 – health / increase PA  Q2 - Do they value the importance of the programme  Q2 – Do they have preconceived goals / ideas  Q2 – Do they know what the referral is, are they given much information from the referring health professional  Q2 – you say that the clients don’t really want to be here, do you have pressure for management to sign them up to long term membership  Q3 – you talk about… (Listen to response) for different clients, can you think of examples where it has worked / not worked.  Q4 – Do clients ask for advice on other exercise / diet / smoking / lifestyle |
| **Descriptive of ERS sessions** | 1. Can you walk me through a typical session you might prescribe? 2. Can you describe the typical setting of a session? 3. Can you describe how you like to deliver a prescription session? 4. Can you talk me through the type of support you offer during a session (observation / supervision / technique)? 5. Can you talk me through what you see as a perfect / gold standard session? 6. Can you talk me through how you determine when your progress / regress a prescription? 7. Do you feel restricted when prescribing? 8. Do participants come in asking to do certain types of exercise 9. Putting yourself in their shoes, do they know what sort of exercises they will be doing? | Can you give me examples of this?  Can you share a time that happened?  Q1/2 – You say there is no typical…, can you elaborate why  Q1 – What is your thinking in why you have opted for that style of lesson  Q1 – You talk about these types of exercise; do you find that the delivery fit in – aerobic / resistance more often – why do you think that might be the case?  Q1/2– You say the client is in control of what they choose.  Q3/4 – you say you mostly observe, is this because clients are generally happy to get on with things – if so, you can talk me through which groups  Q 3/4 – you say you offer techniques / greater supervision, do find certain groups of people ask for more or less  Q5 – How does that differ to what you deliver? Why do you think that is the case?  Q6 – You say you find it hard progressing / regressing. What sort of barriers do you face? - example of how you’ve overcome those barriers  Q7 – how so? |
| **Motivational strategies relating to exercise prescription** | 1. Can you describe your rationale for the starting level of a prescribed session and how you convince a person this is the best place to start? 2. Can you talk me through what motivational strategies you use with clients (if any)? 3. Putting yourself in the client’s shoes, how much anxiety / fear / excitement is there towards the programme. | Can you give me examples of this?  Can you share a time that happened?  Q1 – You say there is no typical start point – why so.  Q1 – You say the clients work with you; do they pick where they want to start  Q2 – you say it is quite diverse, is there a time where it has worked or not worked– can you give me an example?  Q2 – which BCT have you found to be the best – why?  Q2 – Do certain populations require more support as a case- if so, which?  Q2 – you say you don’t openly use / discuss BCT, why is this?  Q3 – How do long does that last for, do certain populations show greater or lesser tendencies? |
| **Your assessment of the situation / measurement / supervisor / delivery** | 1. Can you described to me what you see as successful? 2. Can you describe how you measure adherence to the prescription? 3. Do you ever face adverse events? If so, what kind, and how do you deal with them? | Can you give me examples of this?  Can you share a time that happened?  Q1 – How do you judge that success  Q1 – you mention seeing clients maintain exercise as success, are memberships conversion important to you / company?  Q3 – e.g. patients just doing their own thing, patients are injured, your choice of activity just not working. |
| **External to ERS** | 1. Do you prescribe activities away from the programme, such as home activities or refer to other programmes? 2. Is there anything else you would like to bring up that you feel I might have missed, or you want to talk more about? | Can you give me examples of this?  Can you share a time that happened?  Q2 – how would you improve the concept exercise referral, what would make your life easier / achieving more with participants? |
